# Directionally tunable co- and counterpropagating photon pairs from a nonlinear metasurface

**DOI:** 10.1515/nanoph-2024-0122

**Published:** 2024-06-25

**Authors:** Maximilian A. Weissflog, Jinyong Ma, Jihua Zhang, Tongmiao Fan, Shaun Lung, Thomas Pertsch, Dragomir N. Neshev, Sina Saravi, Frank Setzpfandt, Andrey A. Sukhorukov

**Affiliations:** 9378 Institute of Applied Physics, Abbe Center of Photonics, Friedrich Schiller University Jena, 07745 Jena, Germany; Max Planck School of Photonics, Hans-Knöll-Straße 1, 07745 Jena, Germany; ARC Centre of Excellence for Transformative Meta-Optical Systems (TMOS), Department of Electronic Materials Engineering, Research School of Physics, The Australian National University, Canberra, ACT 2600, Australia; Songshan Lake Materials Laboratory, Dongguan, Guangdong 523808, P.R. China; Fraunhofer Institute for Applied Optics and Precision Engineering IOF, Albert-Einstein-Straße 7, 07745 Jena, Germany

**Keywords:** SPDC, nonlinear metasurface, photon pairs, tuning, guided-mode resonance, spatial control

## Abstract

Nonlinear metasurfaces have recently been established as a new platform for generating photon pairs via spontaneous parametric down-conversion. While for classical harmonic generation in metasurfaces a high level of control over all degrees of freedom of light has been reached, this capability is yet to be developed for photon-pair generation. In this work, we theoretically and experimentally demonstrate for the first time precise control of the emission angle of photon pairs generated from a nonlinear metasurface. Our measurements show angularly tunable pair generation with high coincidence-to-accidental ratio for both co- and counterpropagating emission. The underlying principle is the transverse phase matching of guided-mode resonances with strong angular dispersion in a nonlinear metasurface consisting of a silicon dioxide grating on a nonlinear lithium niobate guiding layer. We provide a straightforward design strategy for photon-pair generation in such a device and find very good agreement between the calculations and experimental results. Here, we use all-optical emission angle tuning by means of the pump wavelength; however, the principle could be extended to modulation via the electro-optic effect in lithium niobate. In sum, this work provides an important addition to the toolset of subwavelength thickness photon-pair sources.

## 1 Introduction

Nonlinear nanostructures and metasurfaces enable the generation of light with tailored properties in an ultra-compact, flat form factor [1]. In the classical regime, control over various degrees of freedom such as the generated frequency [2], polarization state [3], optical wavefront [4], [5], and orbital angular momentum [6] have been realized for various harmonic generation processes in nonlinear nanostructures. With the emergence of quantum-entangled photons as key-enabler for applications like secure communication protocols [7] or quantum-enhanced imaging and sensing [8], leveraging the ability of metasurfaces to control nonlinearly generated light has great potential [9], [10], [11], [12], [13]. Indeed, recently the generation of photon pairs using spontaneous parametric down-conversion (SPDC) has been observed in nonlinear metasurfaces [14], [15], [16], [17] as well as isolated nanoparticles [18], [19], [20]. First demonstrations included the control of the spectrum of down-converted photons. The usually broad spectrum of photon pairs from non-phase-matched, thin nonlinear crystals [21], [22] can be reshaped and controlled by introducing resonances via nanostructuring [14], [15]. Furthermore, thin-crystal based SPDC sources allow the generation of various polarization entangled quantum states [23] and also the tuning of the degree of polarization entanglement [24], a concept that can be extended when leveraging resonances in nanoresonators [25].

**Figure 1:**
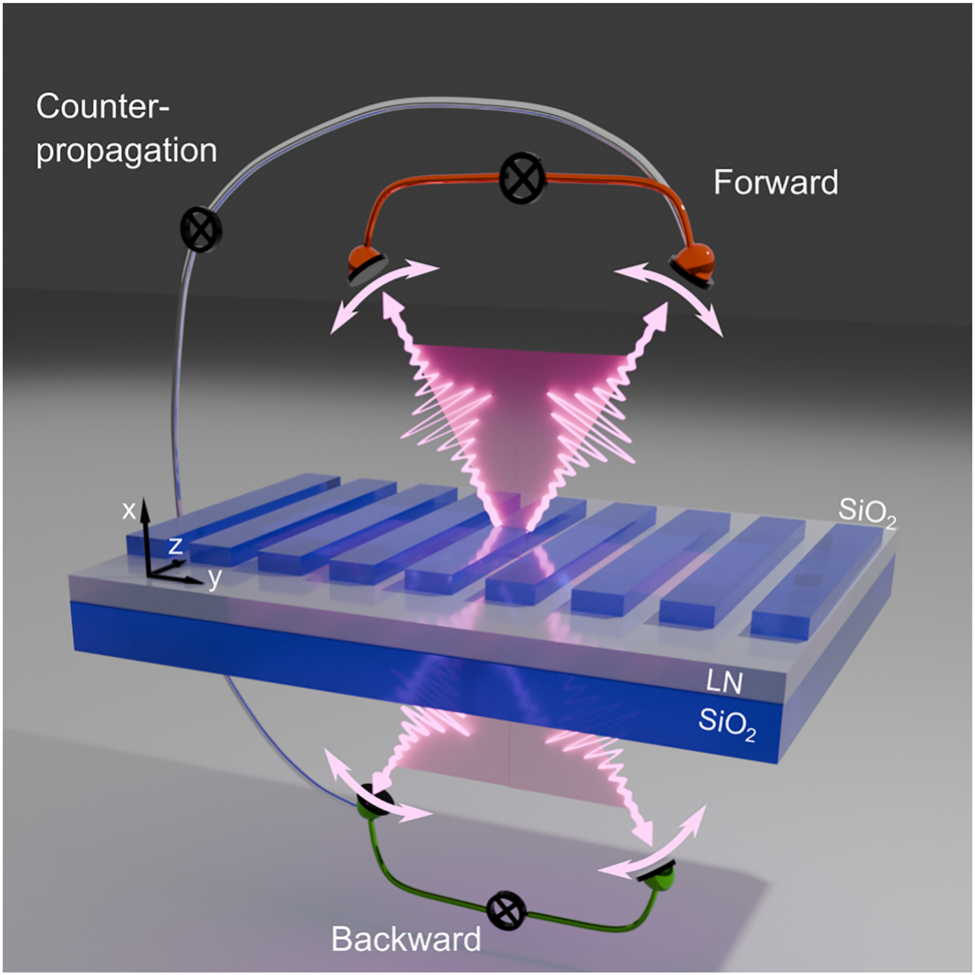
Principle of a metasurface that generates angularly tunable photon pairs in co- and counterpropagating geometry.

However, many capabilities of nonlinear nanostructures demonstrated in the classical regime are yet to be developed for quantum processes in metasurfaces. An example is the control of the spatial emission direction: for classical frequency up-conversion, various concepts for flexible tuning of the free-space emission direction exist [26]. In contrast, while nonlinear metasurfaces based on Mie-type or quasi-BIC resonances can generate photon pairs in different spatial configurations like backward [14], forward [15], and counterpropagating [16] emission, potentially with high directionality [27], no ability to actively control the spatial properties was demonstrated experimentally. In this work, we close this gap by theoretically and experimentally demonstrating a nonlinear metasurface that offers precise control over the free-space emission direction of photon pairs, see the conceptual sketch in Figure 1. Our metasurface leverages transverse phase matching of guided-mode resonances with strong angular dispersion [28] for efficient pair generation [17], [29], [30]. We further show that this tuning mechanism works both for co- and counterpropagating photon pairs. Importantly, on the conceptual side, we present in this work a straightforward, largely analytical design strategy for a nonlinear metasurface that generates spatially tunable co- and counterpropagating photon pairs. The experimental results are in very good agreement with our analytic predictions. On the practical side, our photon pair source reaches a coincidence-to-accidental ratio (CAR) of 7,500 for counterpropagating pair generation, a very high value for the class of (sub-) wavelength thickness sources [14], [15], [16], [19], [20], [21], [23], [31]. This is an important step toward more practically usable nanoscale pair sources, which generally suffer from a high noise level due to photoluminescence and, therefore, low state purity [32].

## 2 Results & discussions

### 2.1 Metasurface design for directionally tunable photon-pair generation

Generally, in photon-pair generation via SPDC, a pump photon with frequency *ω*_*p*_ splits into signal and idler photons with frequencies *ω*_*s*,*i*_, where the energy is conserved such that *ℏω*_*p*_ = *ℏω*_*s*_ + *ℏω*_*i*_. In this work, we consider a metasurface with subwavelength thickness, where the longitudinal phase-matching condition is relaxed [21]. The transverse pump momentum in the transversely extended structure will be conserved by the signal and idler momenta, i.e., $k_{p}^{⊥}=k_{s}^{⊥}+k_{i}^{⊥}$ , where $k_{n}^{⊥}=\left(k_{y,n},k_{z,n}\right)$ and *n* = *p*, *s*, *i* indicates the pump, signal, or idler photon.

The main objective of this work is twofold: the design of a nonlinear metasurface that can both generate photon pairs highly tunable in their emission angle and emit them into a copropagating and also counterpropagating configuration. For this, a nonlinear device that shows high angular dispersion and allows photon-pair emission into the same or two opposing half-spaces (air side or substrate side) is needed.

**Figure 2:**
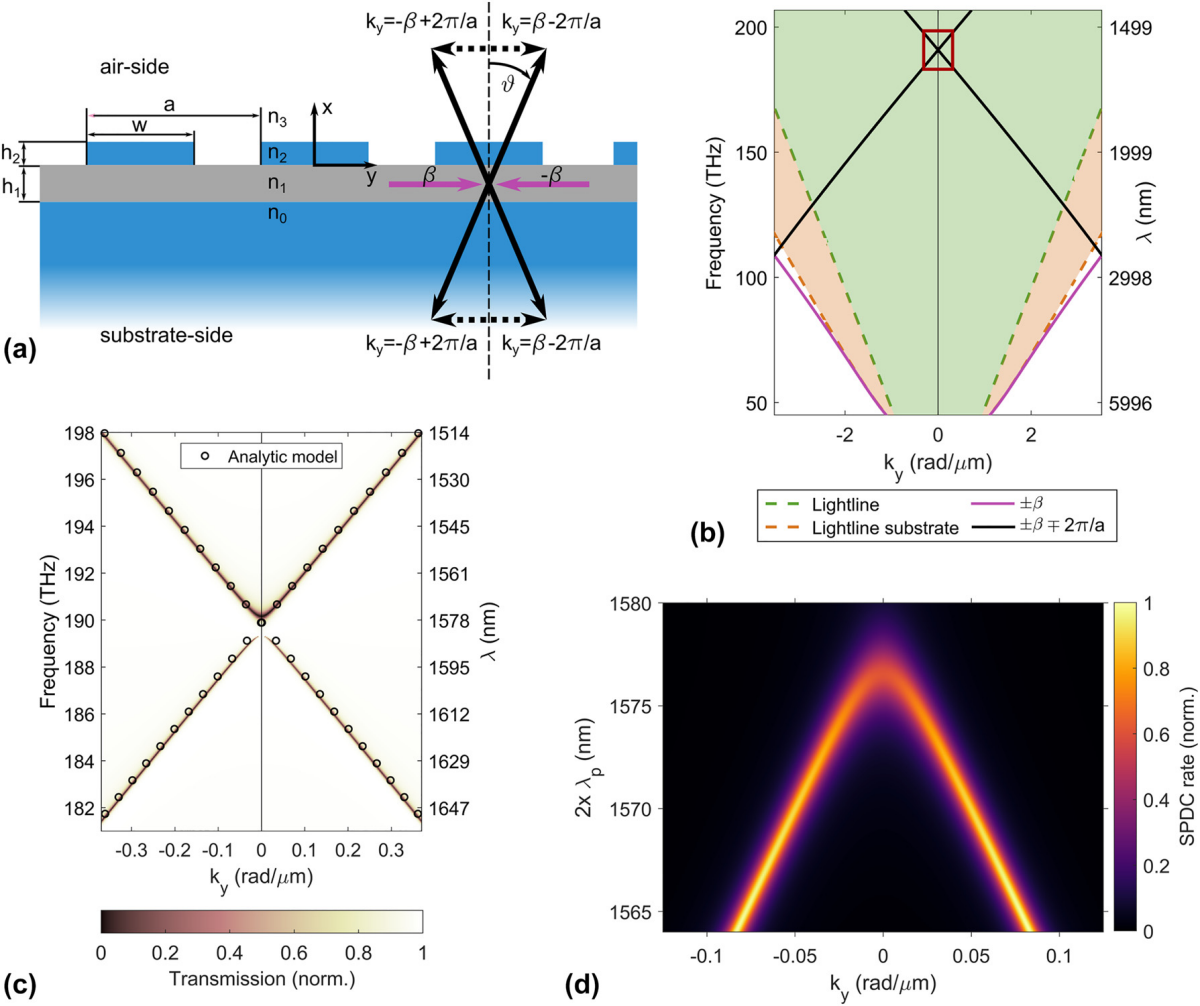
Metasurface supporting guided-mode resonances. (a) Schematic of the geometry of the employed metasurface consisting of a lithium niobate thin film (gray) on a silicon dioxide (SiO_2_, blue) substrate and covered by a SiO_2_ grating. The geometrical parameters are *h*_1_ = 308 nm, *h*_2_ = 210 nm, *w* = 560 nm, and *a* = 892 nm. The metasurface supports counterpropagating modes with propagation constant ±|*β*|, which can couple to free-space modes with transverse wavevector *k*_*y*_ under a propagation angle *ϑ* via the additional momentum provided by the grating. (b) Band structure of TE modes in the meta-grating. The ±1st orders (black solid lines) are above the light line for both the air and substrate side (green and orange dashed lines and shaded areas, respectively) and, therefore, allow photon emission into both half-spaces. (c) Zoom-in to the region of the bandgap at *k*_*y*_ = 0. The analytically calculated band dispersion, assuming a vanishing index modulation (black circles), is overlaid with the numerically computed transmission spectrum using rigorous coupled wave analysis (RCWA) for the finite index modulation. (d) Numerical simulation of the evolution of photon pair transverse wavenumber that defines the emission angle for varying signal and idler wavelength *λ*_*s*_ = *λ*_*i*_ = 2 × *λ*_*p*_.

We will first analytically demonstrate that a nonlinear metasurface consisting of a grating structure coupled to a nonlinear slab-waveguide perfectly combines both these conditions. We use a similar base design as in [17], a silicon dioxide (SiO_2_) grating with period *a*, duty cycle *DC* = *w*/*a*, and height *h*_2_ on top of an *x*-cut nonlinear lithium niobate (LN) waveguide with height *h*_1_ (see Figure 2(a)). LN is a well-suited material for quantum-optical applications at the nanoscale due to its high second-order nonlinearity, large optical transparency window, low fluorescence, and the availability of high-quality LN thin films on low-index substrates [5], [33]. The cladding is air; therefore, *n*_3_ = 1. The linear transmission through such a device exhibits sharp resonances, so-called guided-mode resonances (GMR) [28], [34], which occur whenever an impinging lightwave is coupled to a waveguide mode. Accordingly, if a pair of signal and idler photons is generated via SPDC in the nonlinear waveguide layer, we can leverage such GMRs to couple them out into a free-space mode propagating under a specific angle.

Let us first consider a weak grating, where the index modulation Δ*n* = *n*_2_ − *n*_3_ → 0 vanishes. Although this is not strictly the case for a SiO_2_ grating with air cladding, this approximation allows us to analytically predict the emission angle of the photon pairs by finding a solution for the angular dispersion of the GMR resonances [28], [34]. In the weak grating limit, the propagation constant *β* of a guided mode can be calculated analytically by approximating the grating region as a homogeneous layer with averaged refractive index *n*_avg_ = *DC* × *n*_2_ + (1 − *DC*) × *n*_3_ [35]. Note that in our *x*-cut LN film, the largest LN nonlinear tensor component $\chi_{\mathrm{zzz}}^{\left(2\right)}$ mediates down-conversion of a *z*-polarized pump photon into a pair of *z*-polarized signal and idler photons. This corresponds to TE polarization in the multilayer waveguide with *j* = 1, …, *N* layers. The characteristic equation for TE-modes is [36]

(1) $$1=-\frac{q_{N}}{q_{N+1}}\tanh\left(q_{N}h_{N}+\psi_{N}\right),$$

which is derived from the transverse boundary condition between the *N*th layer and the cladding. It contains the transverse wave-vector $q_{j}=\sqrt{\beta^{2}-{\left(2\pi /\lambda \right)}^{2}n_{j}^{2}}$ , the free-space wavelength *λ*, the refractive index *n*_*j*_, and the phase tanh(*ψ*_*j*+1_) = *q*_*j*_/*q*_*j*+1_ tanh(*q*_*j*_ *h*_*j*_ + *ψ*_*j*_) for all *j* = 1, …, *N* layers. Based on the boundary condition for the initial phase tanh(*ψ*_1_) = *q*_0_/*q*_1_, the solution of Eq. (1) is straightforward. Refer to section S1 of the Supplementary Material for more details. For any mode with propagation constant *β* that solves Eq. (1), a counterpropagating mode with propagation constant −*β* exists, see the purple arrows in Figure 2(a) and the purple graphs in Figure 2(b). A photon propagating in the mode ±*β* can be coupled out of the waveguide if its transverse momentum is above the light line of the respective half-space. Since we are aiming for a source of counterpropagating photons along ±*y* in free space, out-coupling both to the air side and the substrate side needs to be possible. The light lines for free space and the substrate are marked with green and orange dashed lines, respectively, in Figure 2(b). We choose now a set of parameters for waveguide and grating constant, such that the bands corresponding to the first grating orders *β* − 2*π*/*a* and −*β* + 2*π*/*a* cross *k*_*y*_ = 0 close to the telecom wavelength range at ≈1,570 nm. The analytically calculated band structure for TE modes assuming a vanishing index modulation Δ*n* → 0 is shown in Figure 2(b).

For finding the accurate size of the bandgap at the mode crossing point *k*_*y*_ = 0 for a finite index modulation, we use rigorous coupled wave analysis (RCWA) [37] to simulate the transmission spectrum for incident plane waves, see the zoomed-in Figure 2(c). The very good agreement between the weak grating approximation and the rigorous calculation underpins that the analytical model is indeed an excellent guide for analyzing the angular dispersion of the metasurface. Note that at *k*_*y*_ = 0, the transmission line at the branch below the bandgap vanishes (long wavelength side). This is because the corresponding eigenmode at *k*_*y*_ = 0 has an antisymmetric electric field profile that does not couple to an incident plane wave. In this work, all measurements will be performed in the branch above the bandgap (short wavelength side).

The dispersion relations in Figure 2(b) and (c) further demonstrate that out-coupling to both sides and, therefore, counterpropagating pair emission is possible. The transverse wave vector $k_{s,i}^{⊥}$ of the signal and idler photon directly depends on the propagation constant *β*_*s*,*i*_ of the guided modes and thus on the signal/idler frequencies *ω*_*s*,*i*_. We use a normally incident pump beam with zero transverse momentum $k_{p}^{⊥}=0$ , which means the signal and idler transverse momenta have to satisfy *k*_*y*,*s*_ = −*k*_*y*,*i*_. Given the mode dispersion diagram in Figure 2(b), this condition, as well as energy conservation, is satisfied for two frequency degenerate, counterpropagating modes ±*β*. Therefore, we can select the emission angle of the photon pair all-optically by tuning the pump frequency *ω*_*p*_ = 2 × *ω*_*s*,*i*_, controlling in which two counterpropagating modes the signal and idler photons will be generated.

After these linear design considerations, we proceed to a calculation of the nonlinear down-conversion process. For highly multimodal, nanoscale devices, modeling the quantum process of a spontaneous decay into signal and idler photon is most efficiently treated using a Green’s function approach [18], [25], [38]. However, for a metasurface supporting only one mode in the frequency region of interest and the further restriction to frequency degenerate SPDC with *ω*_*s*_ = *ω*_*i*_, the quantum-classical correspondence between sum-frequency generation and SPDC [9], [39], [40] can be efficiently used together with coupled-mode theory, to model the down-conversion process [17]. The basic idea of quantum-classical correspondence is to infer the probability of down-conversion from a certain pump mode characterized by $\left\{\omega_{p},{\vec{k}}_{p}\right\}$ into two signal/idler modes $\left\{\omega_{s,i},{\vec{k}}_{s,i}\right\}$ , by calculating the efficiency of classical frequency up-conversion from $\left\{\omega_{s,i},-{\vec{k}}_{s,i}\right\}$ to $\left\{\omega_{p},-{\vec{k}}_{p}\right\}$ . As a validation of our metasurface design for angularly tunable SPDC, we calculate the down-conversion probability via the correspondence principle for varying signal/idler emission angles toward the air side with pump incidence from the substrate side considering frequencies *ω*_*p*_ = 2 × *ω*_*s*,*i*_, see Figure 2(d). We confirm that the emission angles of the photon pairs closely follow the dispersion of the GMRs. Please note that Figure 2(d) is plotted in a linear wavelength scale and not frequency to facilitate a comparison with the experimental results presented in the next section.

### 2.2 Experimental characterization of co- and counterpropagating pair generation

**Figure 3:**
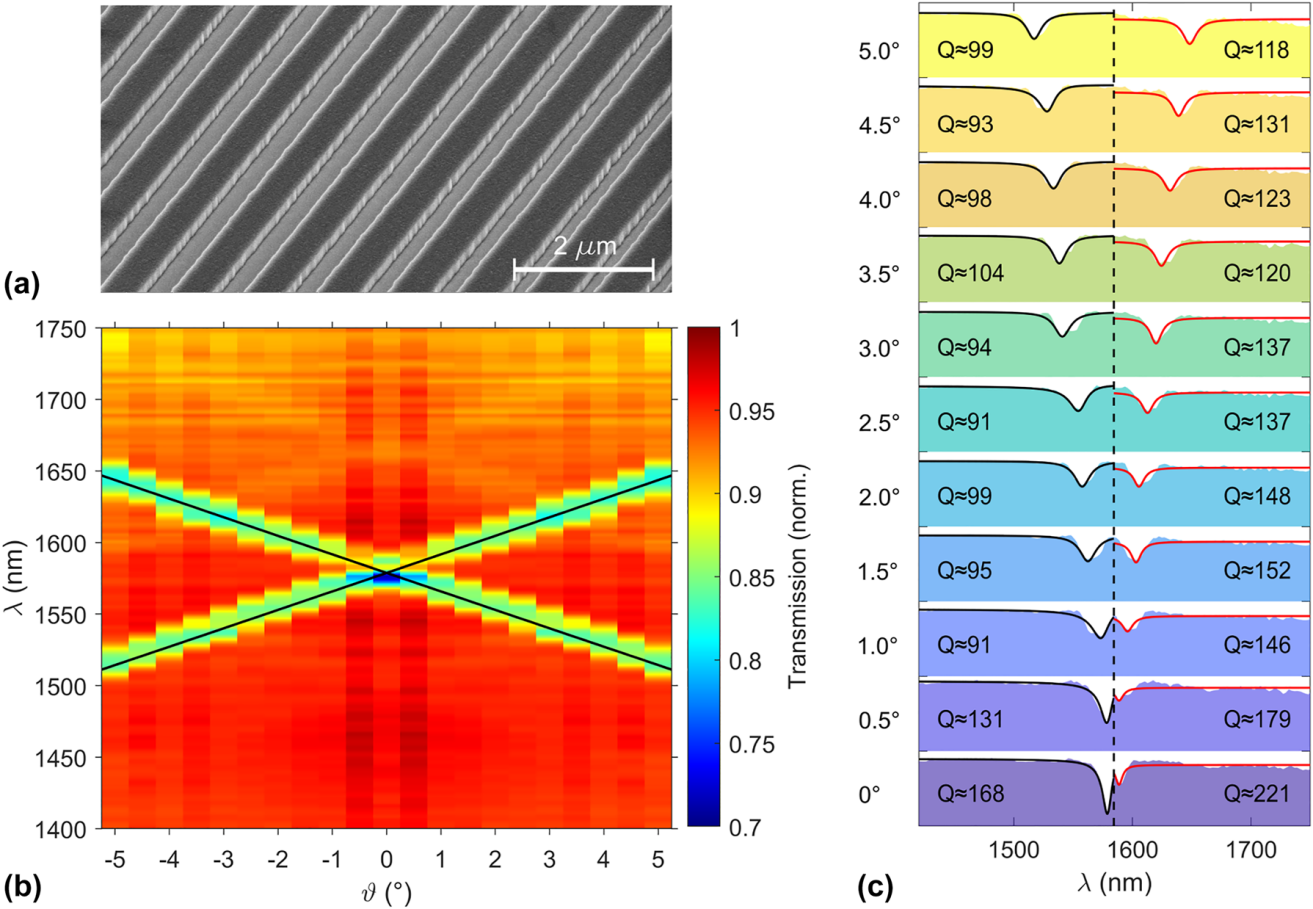
Linear characterization for varying incidence angles. (a) Scanning electron micrograph of the fabricated metasurface. (b) Measured transmission spectrum of the metasurface in the telecom wavelength range for different incidence angles. Guided-mode resonances (GMR) are clearly appearing as minima in the transmission spectra, the bandgap is located at *λ*_gap_ = 1,581 nm. The dispersion of the GMR closely follows the prediction based on the analytical model (black lines). Note that the measurements were carried out for incidence angles 0° ≤ *ϑ* ≤ 5°. For the sake of better illustrating the band structure, they are plotted mirror-symmetric, also including negative incidence angles. (c) Fits with Fano line shapes to the measured linear spectra for varying incidence angles. Shaded areas are the measurement, where the baseline for all plots is set at 65 % transmission. Black solid lines are the Fano fit to the short-wavelength resonance, and red solid lines are the Fano fit to the long-wavelength resonance. The black dashed line marks the division between the fitting regions. The *Q*-factor calculated based on the Fano fit is indicated for each resonance.

We fabricate the designed nonlinear metasurface by deposition of SiO_2_ on a commercial LN thin film with quartz substrate [33] and subsequent patterning of a 400 μm × 400 μm sized grating structure by etching of the SiO_2_ layer only [17]. While the fabrication of grating structures by directly patterning LN is possible [41], creating steep side walls and low surface roughness in LN nanostructures is in general challenging [33]. The approach used here without patterning LN, therefore, facilitates fabrication and keeps scattering losses in the LN waveguiding layer at a minimal level. Figure 3(a) shows a scanning electron micrograph of the fabricated structure. The linear properties of the metasurface are probed by transmission spectroscopy, see Figure 3(b). For broadband illumination with a thermal lamp, pronounced dips in the transmission spectrum are visible, which spectrally shift with varying incidence angles *ϑ*. The upper and lower branches of the modes, as well as the bandgap, are clearly visible. Overlaying the transmission plot with the analytically calculated mode dispersion confirms a very good agreement between the theoretically expected and experimentally obtained linear device properties. Note that for the calculation, we use refractive index data measured by ellipsometry directly on our sample.

We further fit Fano line shapes to the experimentally measured transmission spectra, see solid lines in Figure 3(c), and calculate the *Q*-factor of the resonances in the fabricated device (see Supplementary section S4 for details of the Fano fits). The experimentally measured *Q*-factor for the short-wavelength resonance close to the bandgap is *Q* ≈ 168 and, at off-normal emission angles, slightly reduces to *Q* ≈ 90–100. The observed stability of the *Q*-factor over an extended angular range is a desirable property for directional tuning since it ensures efficient photon pair generation under various angles. Note that the modulation depth and *Q*-factor of the resonances observed in the transmission measurement are in part limited by the resolution of the available spectrometer (Ocean Insight NIRquest512 with full width at half maximum resolution of 3.1 nm). Based on our simulations, we expect that the actual *Q*-factor is in fact above the values measured in this experiment, which is in-line with results from previous work [17].

**Figure 4:**
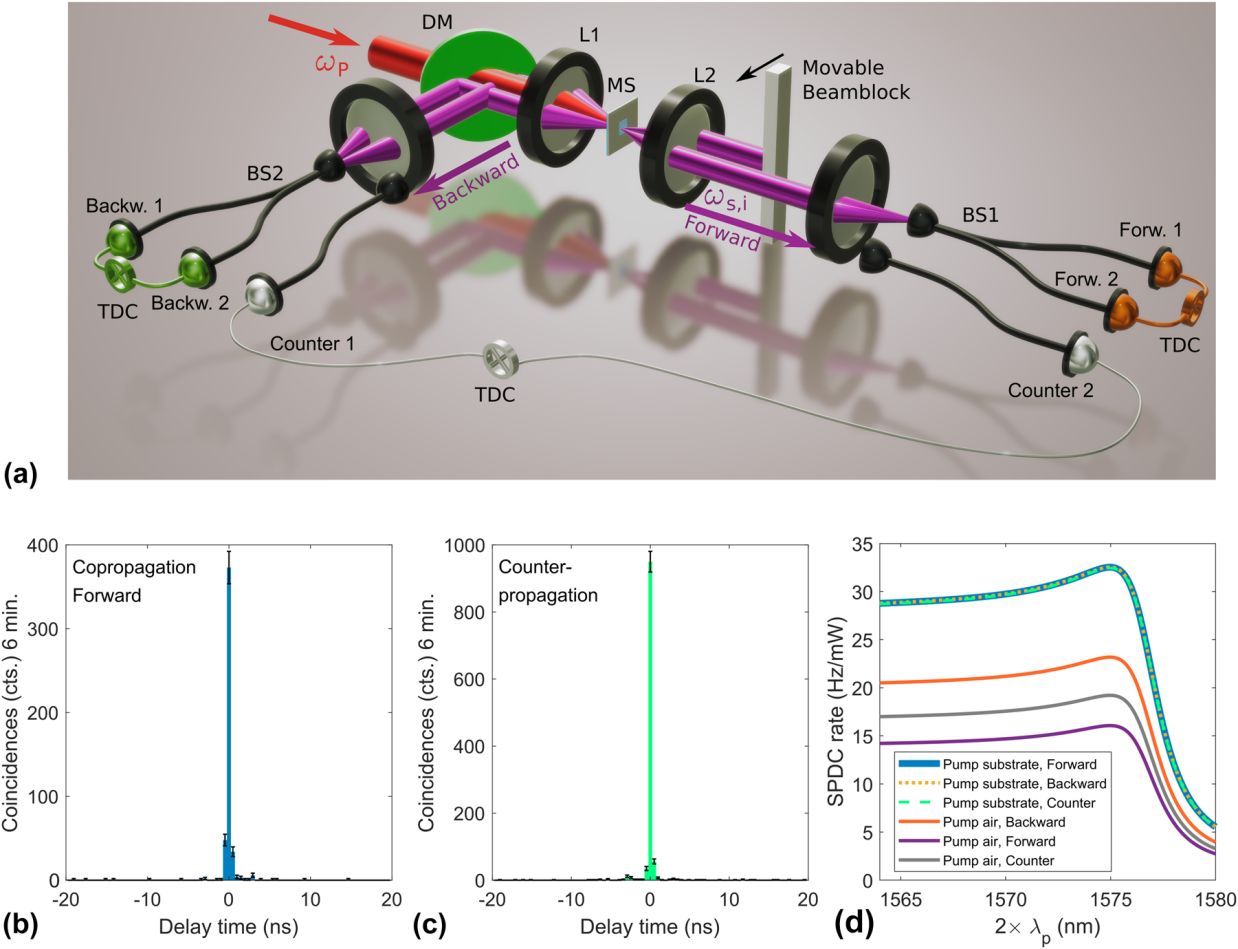
Photon-pair generation in co- and counterpropagating geometry. (a) Schematic of the Hanbury Brown–Twiss interferometer used for coincidence measurements. A continuous-wave pump laser tunable in the range 782.7 nm ≤ *λ*_*p*_ ≤ 789.5 nm is focused onto the metasurface by lens L1 (*f* = 100 mm). Photon pairs in the forward direction are collected by lens L2 (*f* = 50 mm), while pump photons are removed using a combination of interference filters (not shown). SPDC photons propagating in the backward direction are separated from the pump photons using a dichroic mirror (DM). For copropagating collection geometries in the forward direction (detector position Forw. 1 & 2, orange) or backward direction (detector position Backw. 1 & 2, green), nonpolarizing multimode fiber-beamsplitters are used. For counterpropagating detection geometry (detector position Counter 1 & 2, white), the photons are automatically separated. Temporal delays are measured using a time-to-digital converter (TDC). A movable beam block positioned in the back-focal plane of L2 allows the characterization of the propagation direction of photons in the transmission arm. (b)–(c) Coincidence histograms for (b) copropagating forward and (c) counterpropagating detection geometry and excitation incident from the substrate side. The excitation power was *P* = 91 mW at *λ*_*p*_ = 785 nm and the exposure time *t* = 6 min. Error bars mark the statistical uncertainty. (d) Simulated SPDC rate for varying pump wavelengths, excitation, and detection geometries.

For the characterization of generated photon pairs, we use a Hanbury Brown–Twiss setup, see Figure 4(a), where pair generation is detected by temporally coincident excitation of two multimode fiber-coupled single-photon avalanche diodes (SPADs). As a pump source, a tunable continuous-wave diode laser is used. In order to measure pair generation in co- or counterpropagating emission configurations, correlations between the signals from the detectors in different positions are measured as marked in Figure 4(a). For pump photon suppression, we use a combination of interference long-pass filter (cut-on wavelength 1,150 nm) and a band-pass filter (central wavelength 1,575 nm, bandwidth 50 nm) in forward and backward path. In sections S2 and S3 of the Supplementary Material, we provide more details on the experimental setup as well as a characterization of the pump laser.

We perform correlation measurements in copropagation (forward) and counterpropagation geometry by exciting the metasurface with a pump beam of *λ*_*p*_ = 785 nm and power *P* = 91 mW incident from the substrate side, compare the coincidence histograms in Figure 4(b) and (c) (error bars mark the statistical uncertainty). We find for the emission of both photons in copropagation and remarkably also for counterpropagating pair emission a high coincidence rate, marked by sharp correlation peaks around the zero-time delay bin. The coincidence rates *R*_*c*_ extracted from the histograms are *R*_*c*,co_ = 1.28 Hz ± 0.06 Hz for copropagating emission in forward geometry and *R*_*c*,counter_ = 2.92 Hz ± 0.09 Hz in counterpropagating geometry, respectively. The metasurface also generates a sizeable amount of photon pairs in the copropagating backward direction, and an exemplary histogram is found in Supplementary Material section S5 where we also show the power dependence of the coincidence rate. Due to the technical limitations of our current experimental setup, we cannot characterize the angular properties of copropagating photon pairs in the backward direction, and we don’t consider this configuration in the following. We verify by correlation-based fiber spectroscopy [21], [42] that the spectrum of the photon pairs is narrow band and closely follows the linear resonance of the metasurface. See Supplementary section S6 for details.

Of interest is now the ratio of the true photon pair rates *R*_*c*,true_ emitted in the different configurations. This can be estimated from the experimentally observed rate by accounting for the collection and detection efficiencies in both detection paths *η*_forw∕backw_. However, in the case of a subwavelength thickness photon-pair source, the absolute calibration of these efficiencies proves to be difficult. The presence of noise, e.g., from photoluminescence in LN [21], [43], inhibits the estimation of the collection efficiency via a simple calculation of the heralding efficiency [32]. Therefore, we measure the transmission and collection efficiencies of a classical Gaussian beam at 1,575 nm, leading to estimated single photon collection efficiencies of *η*_forw_ ≈ 47 % in the forward path and *η*_backw_ ≈ 20 % in the backward path. The difference between the forward and backward arm is due to the different optical components in both beam paths, see Supplementary Material section S2 for details.

Note that the coupling efficiency measured for a Gaussian beam will generally be considerably higher than for photon pairs generated by the nonlinear metasurface. Depending on the excited guided-mode resonance, pairs are emitted from the metasurface in two lobes with varying angular direction. Therefore, their overlap and correspondingly coupling efficiency to the fiber modes will be reduced as compared to a Gaussian beam. Furthermore, the pair coupling efficiency will vary with emission direction. For these reasons, the collection efficiency of *η*_forw_ ≈ 47 % should be regarded as an upper bound for the used experimental setup and will likely be considerably lower for photon pairs emitted by the metasurface. When accounting also for the quantum efficiency of the single-photon detectors of 25 % and the nondeterministic beamsplitter used only in the copropagating configuration, we estimate the normalized pair rates for the histograms shown in Figure 4(b) and (c) to be in the range of ≈2 Hz/mW for the copropagating forward geometry and about 5 Hz/mW for counterpropagating emission. Since this value is based on the Gaussian beam calibration, it has to be considered a conservative estimate for the photon pairs from the metasurface, which is subject to a large error margin.

We also numerically calculate the generation efficiencies based on quantum-classical correspondence. In Figure 4(d), we show the simulated generation efficiency of degenerate SPDC for different pump wavelengths, collection geometries, and excitation scenarios, i.e., pump incident from air or substrate side. Qualitatively, the pair rate evolves with frequency the same for all excitation and detection scenarios. Away from the bandgap centered at *λ*_gap_ = 1,581 nm, the generation efficiency is only weakly dependent on the pump wavelength and then significantly drops in the bandgap, where the density of optical states is reduced. An important factor is the incidence direction of the pump field. For excitation from the air side, the generation efficiency is reduced as compared to substrate side excitation. In qualitative accordance with this, we obtain lower pair generation rates in the experiment for air side excitation of the metasurface than for the substrate side excitation, see Supplementary Material. The generation rate of copropagating photons in the backward direction is on a similar level as for the forward geometry, see Supplementary Material section S5. In the simulations, we note that the SPDC rate for excitation from the substrate side is almost equal for all three detection scenarios. This is not a general property of excitation from the substrate side but rather appears as a coincidence for our particular metasurface geometry. When varying the geometric parameters which alters the field overlaps between pump and signal/idler mode fields, the relative pair rate in the different detection directions is changed. Generally, the SPDC rate calculated in the simulations is about 6× to 10× larger than the SPDC rate estimated based on the experiment. We assign this largely to the difficulty in absolutely calibrating the collection and detection efficiency. Additionally, also unavoidable fabrication imperfections in the real sample may slightly decrease the device efficiency.

It needs to be highlighted that the CAR of our lithium niobate meta-grating source based on GMRs exceeds the CAR for many previously reported subwavelength thickness crystals and metasurfaces [14], [15], [16], [20], [23], [31] or microscale, non-phase-matched devices [19], [21] by one to two orders of magnitude – in particular given the operation at the relatively high pump power of 91 mW. This is in line with previous results in GMR-based metasurfaces [17] and can be partly assigned to the wider bandgap of LN as compared to, e.g., (aluminum) gallium arsenide or gallium phosphide [5], which reduces photoluminescence for the here used pump wavelengths. Furthermore, the use of phase-matched pair generation using high-*Q* guided-mode resonances in the metasurface is a very effective way to enhance SPDC while maintaining an ultra-thin design.

### 2.3 Directional tuning of photon pairs

In the next step, we demonstrate the ability to tune the emission angle of photon pairs. Since the flux of the photon pairs is too low for direct back-focal plane imaging with a classical camera, we instead use a movable beam block with size 700 μm placed in the back-focal plane of the collimation lens in forward direction (lens L2, see Figure 4(a)). By moving the beam block across the emission lobe of first the signal and then the idler photon, the detected coincidence rate will drop whenever the beam block is partly or fully obscuring the respective beam path. Therefore, a decrease in coincidences marks the emission angle of the photon pairs. Since the beam block is placed in the back focal plane, the lateral position can be directly converted to the transverse momentum of the photon pairs.

**Figure 5:**
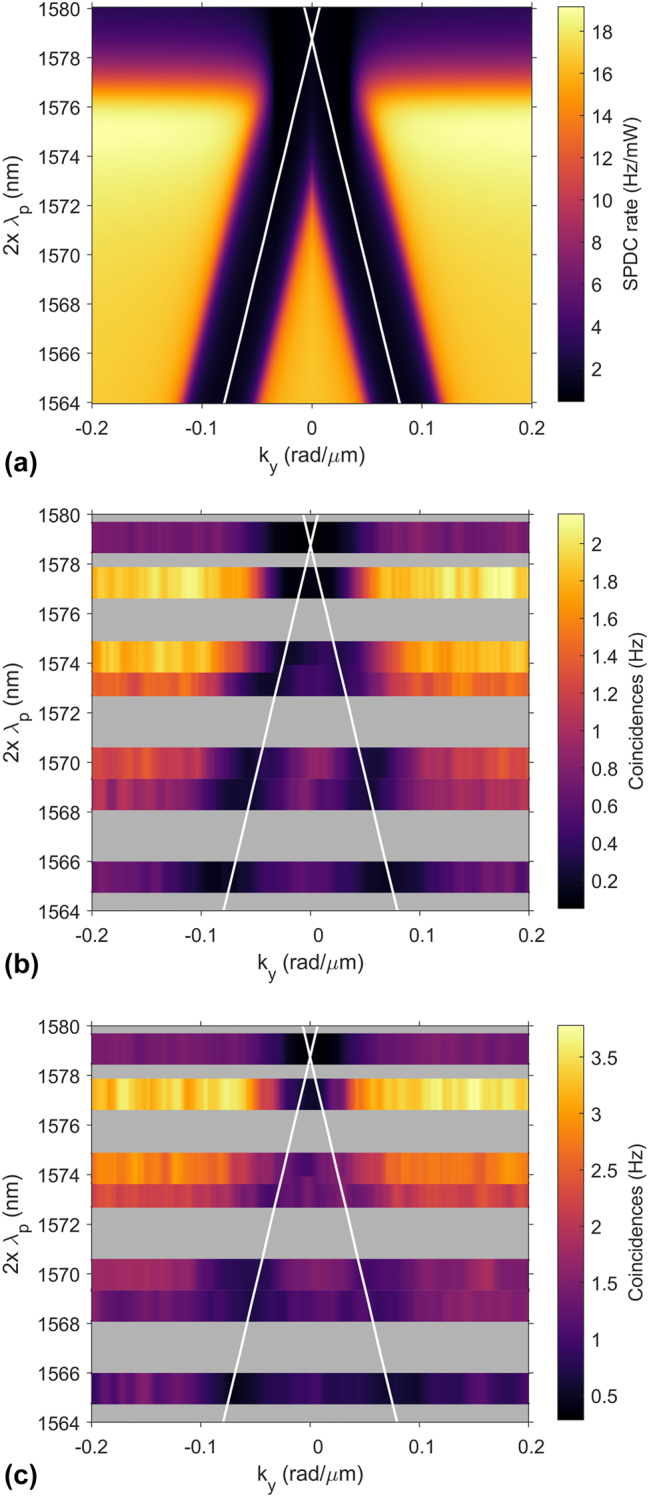
Tuning of photon-pair emission angle. (a) Simulation of SPDC rate in copropagating forward direction depending on the transverse position of a beam block for different degenerate signal and idler wavelengths *λ*_*s*_ = *λ*_*i*_ = 2 × *λ*_*p*_. In this configuration, the beam block will prevent pair-detection whenever it is placed in the emission direction of the signal or idler photon, such that the photon-pair propagation directions are marked by the minima of the detected pair rate. White lines indicate the dispersion of the guided-mode resonances as calculated from the analytical model. (b, c) Experimentally measured coincidence rate in (b) copropagating forward and (c) counterpropagating geometry for different excitation wavelengths and beam-block positions. The emission direction coincides with the transverse momentum of the guided-mode resonances (white lines). Grey bars indicate wavelength regions that are inaccessible due to mode-hopping of the pump lasers.

We use a pump beam incident from the substrate side, which provides the highest pair generation efficiency based on our previous analysis. The expected dependence of the pair generation rate on the beam-block position is shown in Figure 5(a), where the emission angle of the photons increases for a larger detuning from the bandgap at 1,581 nm. The emission direction is identified by clear minima in the pair rate, which appear at angles dictated by the dispersion of the guided modes (see white lines in Figure 5(a)). To accurately resemble the experimental setting, this simulated dependence of the SPDC rate already considers that the measured signal will be a convolution between the SPDC emission pattern in Figure 2(d) and the beamblock function. For the experiment, we use a diode laser tunable in the wavelength range from 782.7 nm ≤ *λ*_*p*_ ≤ 789.5 nm. However, the tunability of the laser is limited by mode hopping, which reduces the number of available pump wavelengths. Wavelength regions that are inaccessible due to mode hopping are marked with gray bars in the experimental plots Figure 5(b) and (c). A detailed characterization of the laser is provided in Supplementary Material section S3.

We measure coincidence histograms for many pump wavelengths and beam-block positions and extract the coincidence counts. In Figure 5(b) and (c), we plot the evolution of the detected coincidence rate with the beam-block position for different excitation wavelengths. Note that we use a moving average filter to partly compensate for fluctuations in the coincidence rate due to the photon statistics. Refer to section S7 of the Supplementary Material for the raw data.

The tuning of the emission angle with pump wavelength is clearly visible. For the copropagating detection configuration in Figure 5(b), two minima in the coincidence rate at opposite transverse wave vectors ±*k*_*y*_ mark the directions where the beam block crosses the emission path of the signal or idler photon. In excellent agreement with the simulation, these emission directions shift to larger angles for decreased pump wavelength. Furthermore, the emission direction of the photon pairs follows the dispersion relation of the GMR frequencies (compare white lines in Figure 5(b) and (c)). We note a reduction of the detected pair rate for larger emission angles, which is somewhat stronger than expected from the simulation. This is mainly due to the reduced fiber-coupling efficiency of the photon pairs in the experiment, which degrades with increased propagation angle.

With these results, we demonstrate that the design of our metasurface is very effective in controlling the spatial properties of the generated photon pairs. Note that in the case of degenerate, copolarized and copropagating SPDC, the definition of signal and idler mode is done based on the spatial propagation direction. The signal photon mode is, e.g., defined to have a wave-vector $-\left|k_{y}\right|=k_{s}$ and the idler photon correspondingly $+\left|k_{y}\right|=k_{i}$ in the same half-space. When turning to a counterpropagating detection geometry, both photons are emitted into opposite half-spaces, which can be used to distinguish signal and idler photons. Now, when carrying out the same emission angle analysis for counterpropagation, see Figure 5(c), we interestingly find two minima again when scanning the beam block. This shows that the single photon emitted into the signal half-space is in a superposition state of propagating along $k_{s}=\pm \left|k_{y}\right|$ . Due to momentum conservation, the idler photon propagates into opposite direction $k_{i}=\mp \left|k_{y}\right|$ , corresponding in total to a path-entangled state $\left|\psi \right\rangle =1/\sqrt{2}\left(\left|\left|k_{y}\right|,-\left|k_{y}\right|\right\rangle +{\text{e}}^{\text{i}\alpha }\left|-\left|k_{y}\right|,\left|k_{y}\right|\right\rangle \right)$ . Due to the grating symmetry with respect of *y* → −*y*, we theoretically expect that *α* = 0 at the degenerate wavelengths of signal and idler photons, although with our experimental configuration, we cannot measure *α*, which would require tomographically complete set of measurements on all paths [44]. There is an interesting potential to tune *α* in the nondegenerate regime and by varying the unit cell symmetry. We envision this to be a promising platform for further investigations, e.g., for the generation of hyper-entangled states by also including the frequency degree of freedom.

## 3 Conclusions

In this work, we designed a nonlinear metasurface consisting of a silicon dioxide grating on a nonlinear lithium niobate guiding layer and experimentally demonstrated photon-pair generation with angular tunability in co- as well as counterpropagating emission. To the best of our knowledge, this is the first demonstration of such precise control over the spatial properties of nonlinearly generated photon pairs from a metasurface. We show that the emission angle of co- and remarkably also counterpropagating photon pairs can be finely tuned by selectively generating signal and idler in guided-mode resonances with high angular dispersion. Moreover, we present a straightforward design strategy for flat, nonlinear SPDC sources that generate tunable and counterpropagating pairs. We, in particular, highlight the latter since generating pairs counterpropagating with respect to the pump direction in a phase-matched nonlinear device is usually a challenging task that requires very careful modal engineering [45] or quasi-phase-matching schemes with very small poling periods [46]. Here, we have shown that by using transversal phase matching of guided modes and probabilistic out-coupling into two opposing half-spaces, counterpropagating pairs can be generated with a very straightforward device design. It is important to note that in our nonlinear metasurface for all detection configurations always, both signal and idler photons are generated on resonance. This is very favorable for the generation efficiency and contrasts with a recent demonstration of counterpropagating pair emission from a BIC-resonant metasurface [16], where one photon of the pair is generated off-resonantly to achieve counterpropagation. In this demonstration, all photon pairs are copolarized along the *z*-direction, following the polarization of the guided modes and the orientation of the largest nonlinear tensor component of LN. By multiplexing several metasurfaces with different orientations, more complex polarization states combined with the spatial tuning degree of freedom can potentially be generated [30]. Going from the here presented 1D periodic to a 2D periodic system also offers additional degrees of freedom for steering photon pairs in more than one plane of incidence, would, however, require additional pump polarization control and a careful optimization of the relative orientation of metasurface lattice and nonlinear tensor [47].

In this work, we control the emission angle all-optically by means of tuning the excitation wavelength. However, since the propagation constant of the guided modes and, therefore, the emission angle sensitively depends on the refractive index of the guiding layer, using the Pockels effect in LN could also be leveraged to electrically modulate the emission direction while keeping the pump wavelength constant. In our experiment, the angular tuning range was only limited by the tunability of the pump laser and the coupling efficiency to the fiber-based detectors. In principle, the underlying physics of guided-mode resonances in our metasurface allows angular tuning in much larger ranges, as already indicated by the linear characterization of the device. Furthermore, the CAR of about 7,500 in counterpropagating geometry is significantly higher than for many other subwavelength thickness crystals or metasurfaces reported so far, which is an important step toward higher practicability of metasurface-based photon pair sources. Nonetheless, for most use cases, the generation efficiency needs to be further increased. This can, for instance, be realized by also resonantly coupling the pump wave to the wave-guiding layer and employing higher-order quasi-phase-matching using periodic poling of the nonlinear metasurface [48] or by optimizing the *Q*-factor of the guided modes [49].

In summary, with this work, we demonstrate precise spatial control of photon pairs generated in a nonlinear metasurface, which adds an important building block to the toolset of subwavelength thickness quantum light sources.

## Supplementary Material

Supplementary Material Details
