# Supplementary material for: Directionally tunable co- and counterpropagating photon pairs from a nonlinear metasurface
Source: Nanophotonics. 2024 Jun 25;13(18):3563–73. doi: 10.1515/nanoph-2024-0122 (PMC11501506; doi:10.1515/nanoph-2024-0122)
Supplement: Supplementary file 1 — Supplementary Material Details [file j_nanoph-2024-0122_suppl_001.pdf]

Maximilian A. Weissflog, Jinyong Ma, Jihua Zhang, Tongmiao Fan, Shaun Lung, Thomas Pertsch, Dragomir N. Neshev, Sina Saravi, Frank Setzpfandt, and Andrey A. Sukhorukov

Supplementary Information Directionally tunable co- and counterpropagating photon pairs from a nonlinear metasurface

Abstract: This Supplementary Information contains 7 sections and 10 figures, providing extra details on the theoretical and experimental aspects of our work.

Contents

S1 Dispersion of guided mode resonance metagrating in weak grating regime — S-2
S2 Experimental setup and estimation of collection efficiencies — S-3
S3 Laser characterization — S-5
S4 Fano-fit parameters for linear transmission measurements — S-5
S5 Comparison of different excitation directions — S-7
S6 Photon-pair spectrum — S-10
S7 Raw data angularly tunable photon-pair generation — S-11

Maximilian A. Weissflog, Institute of Applied Physics, Abbe Center of Photonics, Friedrich Schiller University Jena, 07745 Jena, Germany; Max Planck School of Photonics, Hans-Knöll-Straße 1, Jena, 07745, Germany; maximilian.weissflog@uni-jena.de, https://orcid.org/0000-0002-3091-1441
Jinyong Ma, Tongmiao Fan, Dragomir N. Neshev, Andrey A. Sukhorukov, ARC Centre of Excellence for Transformative Meta-Optical Systems (TMOS), Department of Electronic Materials Engineering, Research School of Physics, The Australian National University, Canberra, ACT 2600, Australia; jinyong.ma@anu.edu.au, https://orcid.org/0000-0001-5753-794X (Jinyong Ma), tongmiao.fan@anu.edu.au (Tongmiao Fan), dragomir.neshev@anu.edu.au, https://orcid.org/0000-0002-4508-8646 (Dragomir Neshev), andrey.sukhorukov@anu.edu.au, https://orcid.org/0000-0002-5116-5425 (Andrey Sukhorukov)
Jihua Zhang, ARC Centre of Excellence for Transformative Meta-Optical Systems (TMOS), Department of Electronic Materials Engineering, Research School of Physics, The Australian National University, Canberra, ACT 2600, Australia; Songshan Lake Materials Laboratory, Dongguan, Guangdong 523808, P.R. China; jihua.zhang@anu.edu.au, https://orcid.org/0000-0002-3506-6638
Shaun Lung, Sina Saravi, Institute of Applied Physics, Abbe Center of Photonics, Friedrich Schiller University Jena, 07745 Jena, Germany; shaun.lung@uni-jena.de, https://orcid.org/0000-0001-7762-6616 (Shaun Lung), sina.saravi@uni-jena.de, https://orcid.org/0000-0003-4089-1189 (Sina Saravi)
Thomas Pertsch, Institute of Applied Physics, Abbe Center of Photonics, Friedrich Schiller University Jena, 07745 Jena, Germany; Max Planck School of Photonics, Hans-Knöll-Straße 1, Jena, 07745, Germany; Fraunhofer Institute for Applied Optics and Precision Engineering IOF, Albert-Einstein-Straße 7, Jena, 07745, Germany; thomas.pertsch@uni-jena.de, https://orcid.org/0000-0003-4889-0869
Frank Setzpfandt, Institute of Applied Physics, Abbe Center of Photonics, Friedrich Schiller University Jena, 07745 Jena, Germany; Fraunhofer Institute for Applied Optics and Precision Engineering IOF, Albert-Einstein-Straße 7, Jena, 07745, Germany; f.setzpfandt@uni-jena.de, https://orcid.org/0000-0002-7919-8181

S1 Dispersion of guided mode resonance metagrating in weak grating regime

Let's consider a GMR grating with a refractive index modulation of the form $n_j = n_{\text{avg}} + f(y)\Delta n$, where n_{avg} is the averaged refractive index of the modulated j^{th} layer. The term $f(y)$ is a periodic function with period a such that $f(y) = f(y+a)$, as it would e.g. apply to the refractive index modulation of the grating shown in Fig. 2(a) of the main text. A rigorous treatment of such guided mode resonance (GMR) gratings requires one to solve the coupled wave-equation for the fields in all modulated or unmodulated layers of the device [1]. However, in case of a weak refractive index modulation $\Delta n \to 0$, the dispersion relation of an unmodulated slab waveguide may be used to predict the propagation constant also of guided modes in the modulated device [2]. In this work, we use this approach to provide an analytical explanation for the angularly tunable photon pair emission. Although in our case the index modulation between silicon dioxide and air in the grating layer is not weak, we still find very good agreement between the analytically calculated resonance positions from the weak-grating model and solutions from rigorous coupled wave analysis (RCWA) [3], see Fig. 2(c) of the main text for a comparison.

In the weak grating limit, the GMR grating can be approximated as a two-layer slab-waveguide, where the grating layer is substituted by a homogeneous layer with averaged index n_{avg}. We use the same nomenclature for all parameters as shown in the sketch of the GMR grating in Fig. 2(a) of the main text. For the solutions of this layered waveguide problem, we follow the approach of [4] and restrict ourselves here to the fundamental TE-mode. The characteristic equation for the multilayer waveguide is

$$1 = -\frac{q_N}{q_{N+1}} \tanh(q_N h_N + \psi_N)\,, \tag{S1}$$

which is derived from the boundary condition between the last guiding layer and the superstrate.

For the two-layer waveguide in this work $N = 2$. The transverse wave-vector q in the different layers is $q_0 = \sqrt{\beta^2 - (2\pi/\lambda)^2 n_0^2}$ for the substrate, $q_1 = \sqrt{\beta^2 - (2\pi/\lambda)^2 n_1^2}$ for the lithium niobate layer, $q_2 = \sqrt{\beta^2 - (2\pi/\lambda)^2 n_{\text{avg}}^2}$ for the grating layer and $q_3 = \sqrt{\beta^2 - (2\pi/\lambda)^2 n_3^2}$ for the superstrate. Here, λ is the free-space wavelength and β is the propagation constant of the guided mode we seek. The phase ψ_j in each layer is determined by the boundary condition for the transverse fields at the layer interface. The initial phase at the substrate interface is

$$\tanh(\psi_1) = \frac{q_0}{q_1} \tag{S2}$$

and the phase condition between the lithium niobate layer and the grating layer

$$\tanh(\psi_2) = \frac{q_1}{q_2} \tanh(q_1 h_1 + \psi_1)\,. \tag{S3}$$

When inserting the relations for $q_{0,\ldots,3}$ as well as (S2) and (S3) into the characteristic equation (S1), we can solve it for the propagation constant β of the waveguide mode. Since Eq. (S1) is a real equation in one variable, the numeric solution is straightforward. For one magnitude of β, two counterpropagating modes with $\pm\beta$ exist.

The momentum contributed by the grating $\beta - 2\pi/a$ and $-\beta + 2\pi/a$, respectively, lifts the dispersion curve of the guided mode above the light line and allows coupling to free-space modes, see the plot of the first Brillouin zone in Fig. 2(b) of the main text.

S2 Experimental setup and estimation of collection efficiencies

The layout of the Hanbury Brown-Twiss setup used for the photon pair correlation measurements is shown in Fig. S1. A continuous-wave (cw) diode laser (Thorlabs FPL785P, see Sec. S3 for a characterization) is sent through an isolator and a dichroic mirror (Thorlabs DMSP1180) and is focused by a plano-convex lens with focal length $f = 100$ mm (Thorlabs LA1509) onto the nonlinear metasurface. The beam diameter at the metasurface is approx. 100 μm. Photons generated via SPDC can propagate in forward and/or backward direction. In forward direction, a plano-convex lens (Thorlabs LA1131-C-ML) is used for collimation and a beamblock with width $w = 700\,\mu$m is placed in its back-focal plane on a piezo stage with closed-loop position sensor (Newport CONEX-SAG-LS16P). To reject pump photons and spurious fluorescence, a short-pass interference filter (Thorlabs FELH1150) and a bandpass filter (Edmund Optics #87-871, center wavelength 1575 nm, bandwidth 50 nm) are used. As detectors, we use two infrared single photon avalanche diodes (IDQuantique ID230) coupled to multimode fibers. Temporal correlations between signals of the two SPADs are registered by a time-to-digital converter (IDQuantique ID801). In the backward direction, SPDC photons are guided towards the fiber coupling point using the dichroic mirror and through an equivalent set of interference filters as in the forward arm.

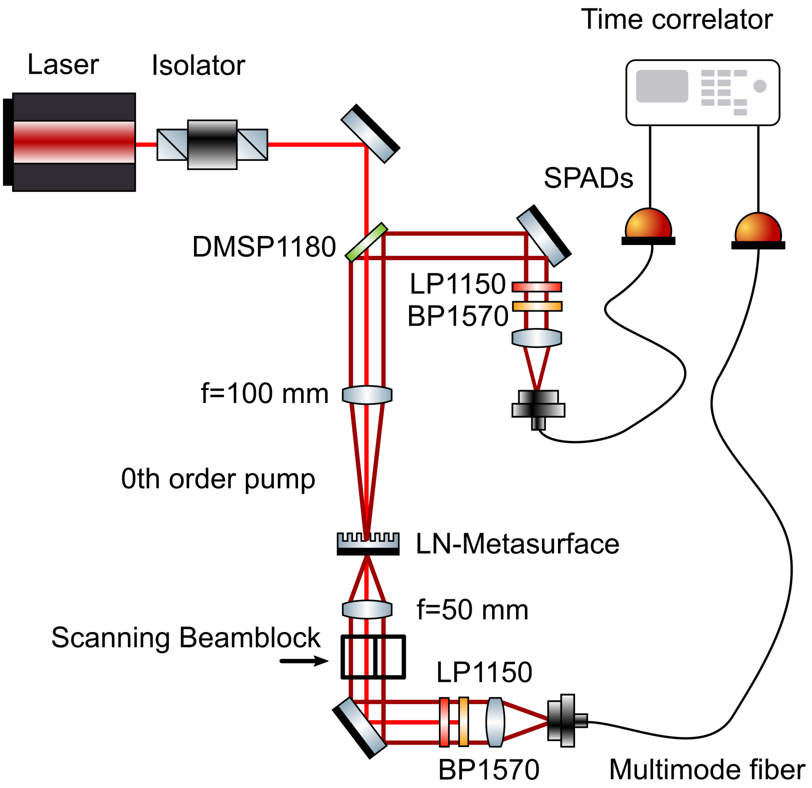

Fig. S1: Layout of experimental setup used for photon pair correlation measurements.

In Fig. S1 the configuration for detecting counterpropagating photon pairs is shown. For characterizing copropagating photons in forward or backward direction, both SPADs are connected via a multimode 50:50 fiber beamsplitter (AFW Techologies) to the forward or backward fiber-coupling point, respectively.

We characterized the collection and detection efficiencies $\eta_{\mathrm{forw,backw}}$ of the optical setup in forward and backward direction using a cw-laser with $\lambda = 1575\,\mathrm{nm}$, close to the degeneracy wavelength of the photon pairs. The optical transmission from the metasurface plane to the fiber-coupling point in forward direction through lenses and filters is $\approx 94\,\%$. Together with a fiber-coupling efficiency of $\approx 50\,\%$, the collection efficiency in forward direction is estimated as $\eta_{\mathrm{forw}} \approx 47\,\%$. Note however, that the coupling efficiency measured for a Gaussian beam will generally be higher than for photon pairs generated by the nonlinear metasurface. As shown experimentally in this work, pairs are emitted from the metasurface in two lobes with varying angular direction. Therefore, their overlap and correspondingly coupling efficiency to the fiber modes will be reduced as compared to a Gaussian beam. Furthermore, the coupling efficiency will vary with emission direction as seen in the drop of the detected rate for larger emission angles in Fig. 5 of the main text. For these reasons, the collection efficiency of $\eta_{\mathrm{forw}} \approx 47\,\%$ should be regarded as an upper bound for the used experimental setup and will likely be considerably lower for photon pairs emitted by the metasurface.

The coincidence detection efficiency in forward direction is then $\eta_{\mathrm{coinc,forw}} = \eta_{\mathrm{forw}}^2 \times \eta_{\mathrm{det}}^2 \times \eta_{\mathrm{BS}} \approx 0.7\,\%$ with SPADs quantum efficiency of $\eta_{\mathrm{det}} \approx 25\,\%$ and the factor $\eta_{BS} = 0.5$ of the probabilistic beamsplitter. We carry out an equivalent analysis in the backward pathway and find $\eta_{\mathrm{backw}} \approx 20\,\%$, where the increased loss compared to the forward path is due to the additional optical components and different coatings. The coincidence detection efficiency in backward direction is $\eta_{\mathrm{coinc,backw}} = \eta_{\mathrm{backw}}^2 \times \eta_{\mathrm{det}}^2 \times \eta_{\mathrm{BS}} \approx 0.1\,\%$. For counterpropagating photon pairs, the coincidence detection efficiency is $\eta_{\mathrm{coinc,counter}} = \eta_{\mathrm{backw}} \times \eta_{\mathrm{forw}} \times \eta_{\mathrm{det}}^2 \approx 0.6\,\%$. Since the photons are here deterministically split by their propagation direction, no beamsplitter is necessary.

S3 Laser characterization

In this section, we characterize the emission spectrum of the tunable diode laser Thorlabs FPL785P, which serves as excitation laser. In Fig. S2(a), the laser spectrum with changing diode temperature is plotted (measured with Ocean Optics QE65000). Note that the plotted spectra are Lorentzian line fits to the measured data points. Mode jumps are clearly visible by discontinuities in the evolution of the spectra. The extracted peak emission wavelengths are shown in Fig. S2(b) and are used for the wavelength calibration for the photon pair emission experiments. Wavelength regions that cannot be reached by the laser due to the mode jumps are marked by grey bars in Fig. 5 of the main text.

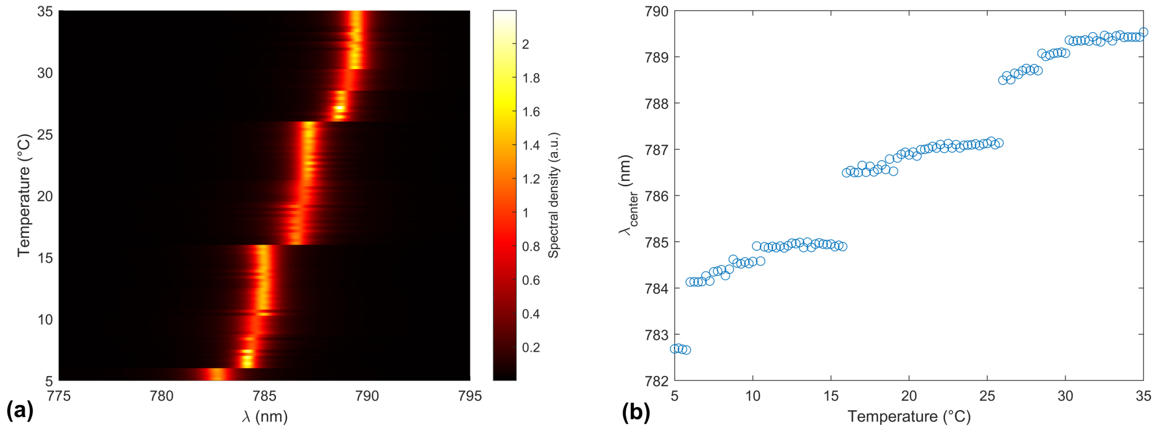

Fig. S2: (a) Spectrum of excitation laser depending on diode temperature. Mode hopping and mode instabilities are clearly marked by discontinuities and instabilities in the evolution of the spectrum. (b) Center wavelength of the excitation laser depending on diode temperature extracted from Lorentzian fits of the measured spectra.

S4 Fano-fit parameters for linear transmission measurements

The linear transmission spectra in Fig. 3(c) of the main text are fit with Fano line-shapes of the form [5], [6]

$$R(\omega) = F\frac{(\epsilon(\omega)+q)^2}{1+\epsilon^2(\omega)} \tag{S4}$$

where $\epsilon(\omega) = 2(\omega-\omega_0)/\Gamma$, q is the Fano (asymmetry) parameter, ω_0 is the resonance frequency and Γ the resonance width at half-maximum. The Q-factor of the resonance is obtained from $Q = \omega_0/\Gamma$. Since we want to fit the Fano shapes to transmission spectra measured in terms of wavelength λ, it is convenient to rewrite eq. (S4) in terms of wavelength,

$$\epsilon(\lambda) = \frac{4\pi c}{\Gamma\lambda\lambda_0}(\lambda_0-\lambda), \tag{S5}$$

where c is the vacuum speed of light and λ_0 the resonance wavelength. For fitting the experimental data, we introduce an additional parameter for the transmission base-line away from the resonance T_0, such that the final fit-formula for the experimental data is

$$T(\lambda) = T_0 - F\frac{(\epsilon(\lambda) + q)^2}{1 + \epsilon^2(\lambda)}. \tag{S6}$$

In Tab. S1 and Tab. S2 we list the parameters for the respective Fano-fits as shown in Fig. 3(c) of the main text. We list these separately for the short wavelength resonance (energies above bandgap, see black graphs in Fig. 3(c) in the main text) and the long wavelength resonance (energies below bandgap, see red graphs in Fig. 3(c) in the main text).

Tab. S1: Fano fit parameters for resonances at short wavelength side (black graphs in Fig. 3(c) of main text).

θ (°)	λ_0 (nm)	ω_0 (THz)	f_0 (THz)	q	Γ (THz)	FWHM (nm)	F	T_0	Q-factor
0	1579.3	1.193E+03	189.83	8.67	7.08	9.38	3.425E-03	0.97	168.44
0.5	1579.3	1.193E+03	189.83	5.71	9.07	12.01	6.019E-03	0.98	131.48
1	1574.5	1.196E+03	190.40	6.20	13.17	17.34	3.531E-03	0.97	90.82
1.5	1562.5	1.206E+03	191.87	203.66	12.67	16.43	2.914E-06	0.96	95.11
2	1557.7	1.209E+03	192.46	119.83	12.23	15.76	8.299E-06	0.96	98.85
2.5	1555.5	1.211E+03	192.73	6.88	13.36	17.16	2.479E-03	0.96	90.66
3	1540.9	1.222E+03	194.56	319.92	13.03	16.42	1.129E-06	0.95	93.83
3.5	1538.5	1.224E+03	194.86	50.88	11.79	14.82	5.036E-05	0.96	103.84
4	1533.9	1.228E+03	195.44	17.03	12.55	15.67	4.294E-04	0.96	97.88
4.5	1529.1	1.232E+03	196.06	6.84	13.17	16.35	2.564E-03	0.96	93.52
5	1517.1	1.242E+03	197.61	232.51	12.57	15.35	2.259E-06	0.95	98.81

Tab. S2: Fano fit parameters for resonances at long wavelength side (red graphs in Fig. 3(c) of main text).

θ (°)	λ_0 (nm)	ω_0 (THz)	f_0 (THz)	q	Γ (THz)	FWHM (nm)	F	T_0	Q-factor
0	1588.7	1.186E+03	188.70	69.19	5.36	7.18	1.932E-05	0.94	221.20
0.5	1588.7	1.186E+03	188.70	77.51	6.63	8.89	1.000E-05	0.95	178.78
1	1595.9	1.180E+03	187.85	86.79	8.06	10.90	9.526E-06	0.93	146.41
1.5	1603.1	1.175E+03	187.01	88.74	7.71	10.52	1.232E-05	0.93	152.40
2	1605.5	1.173E+03	186.73	922.21	7.92	10.84	1.051E-07	0.93	148.13
2.5	1612.7	1.168E+03	185.89	911.33	8.55	11.81	1.157E-07	0.93	136.60
3	1619.9	1.163E+03	185.07	961.35	8.50	11.84	1.299E-07	0.93	136.86
3.5	1624.5	1.160E+03	184.54	888.68	9.68	13.57	1.443E-07	0.93	119.73
4	1631.7	1.154E+03	183.73	1001.49	9.40	13.29	1.039E-07	0.92	122.76
4.5	1638.9	1.149E+03	182.92	1018.74	8.80	12.55	1.066E-07	0.93	130.64
5	1648.3	1.143E+03	181.88	741.18	9.64	13.90	2.108E-07	0.92	118.55

S5 Comparison of different excitation directions

Here, we provide a more detailed analysis of SPDC generation for pump beam incidence either from the substrate or air side. We also show experimental data for copropagating detection in backward direction. Note that these measurements have been carried out on a metagrating with slightly different geometry than the one shown in the main text. The geometrical parameters here are $h_1 = 304\,\mathrm{nm}$, $h_2 = 200\,\mathrm{nm}$, $w = 550\,\mathrm{nm}$ and $a = 890\,\mathrm{nm}$, which result in a band-gap at $\lambda_{gap} = 1573\,\mathrm{nm}$. In Fig. S3 the experimentally measured transmission spectrum of this structure is shown.

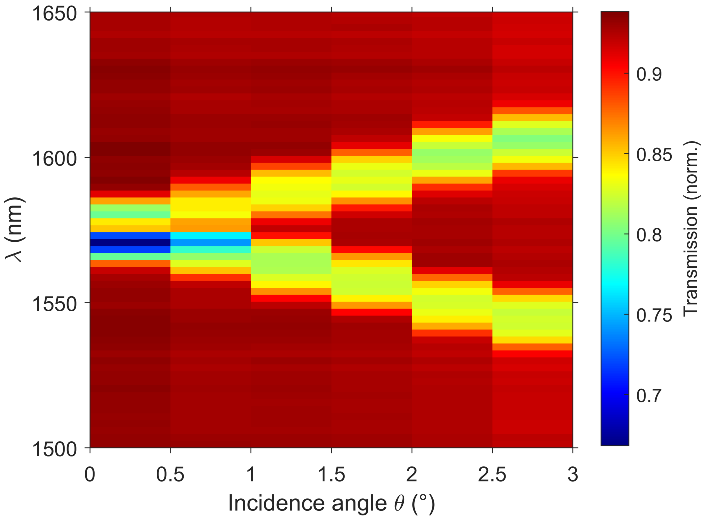

Fig. S3: Measured transmission spectrum of the metagrating used for SPDC measurements as shown in the supplementary information section S5.

In Fig. S4 the measured coincidence rate for the copropagating (forward) and counterpropagating detection is plotted for two different excitation scenarios. The pump wavelength in all cases is 782.7 nm with a power of 92 mW. In Figs. S4(a),(b), the pump beam is incident from the air side, while in Figs. S4(c),(d) the pump beam excites the metasurface from the substrate side. The different incidence directions have here been realized by flipping the sample in the experimental setup.

The extracted pair-rate for substrate side excitation (copropagating (forward) 2.04 Hz± 0.06 Hz, counterpropagating 3.44 Hz±0.08 Hz) is considerably higher than for air side excitation (copropagating (forward) 1.13 Hz ± 0.05 Hz, counterpropagating 1.53 Hz ± 0.06 Hz). Furthermore, the drop in SPDC rate for the counterpropagating detection case is larger than for the copropagating (forward) configuration when switching from substrate to air side excitation. These experimentally observed trends are qualitatively in good agreement with the numerical simulation of the SPDC rate for different configurations, see Fig. 4(d) of the main text. The numerical calculation furthermore predicts, that for substrate side excitation the SPDC rate is the same for all detection configurations. As discussed in the main text and in Sec. S2 this property cannot be tested, due to the difficulty in absolutely calibrating the experimental detection efficiency.

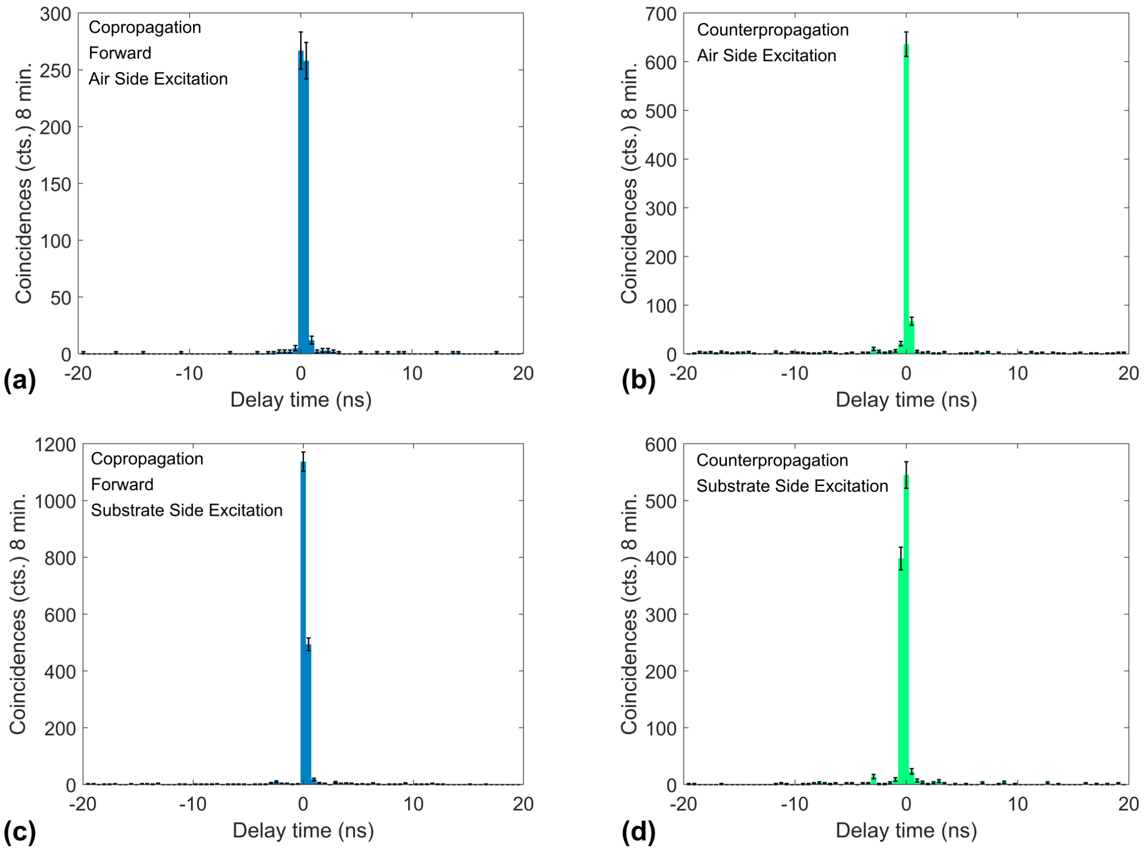

Fig. S4: Coincidence histograms for excitation with $\lambda_p = 782.7\,\mathrm{nm}$, power $P = 92\,\mathrm{mW}$ for different excitation and collection geometries: (a) copropagation (forward) with air side excitation, (b) counterpropagation with air side excitation, (c) copropagation (forward) with substrate side excitation and (d) counterpropagation with substrate side excitation. Error bars mark the statistical uncertainty.

Additionally we measure SPDC in copropagating geometry in the backward direction, see Fig. S5. We find a pronounced coincidence peak, that clearly indicates pair generation in the backward configuration. The observed rate of 0.19 Hz±0.02 Hz is considerably lower than for the other two detection configurations. This is largely due to the higher losses in the backward collection arm, see the discussion in Sec. S2.

When correcting with the estimated collection efficiency for this configuration, the SPDC rate in backward direction is about 1.5 Hz/mW and therefore in a comparable order of magnitude as the other detection cases.

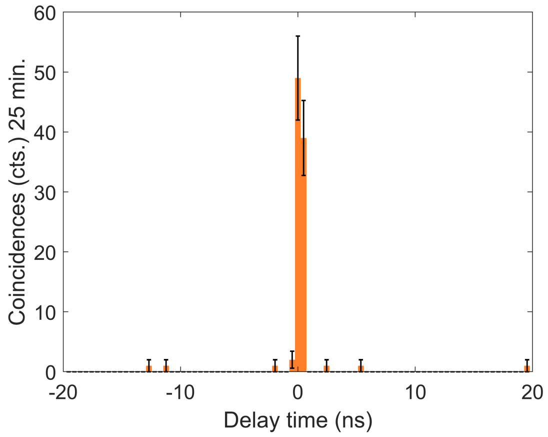

Fig. S5: Coincidence histogram for copropagating (backward) geometry and air side excitation with $\lambda_p = 782.7\,\mathrm{nm}$, power $P = 92\,\mathrm{mW}$. Error bars mark the statistical uncertainty.

We additionally measure the evolution of the SPDC rate with pump power in copropagating forward direction, see Fig. S6. The SPDC rate linearly depends on the pump power, as expected for pair generation via SPDC.

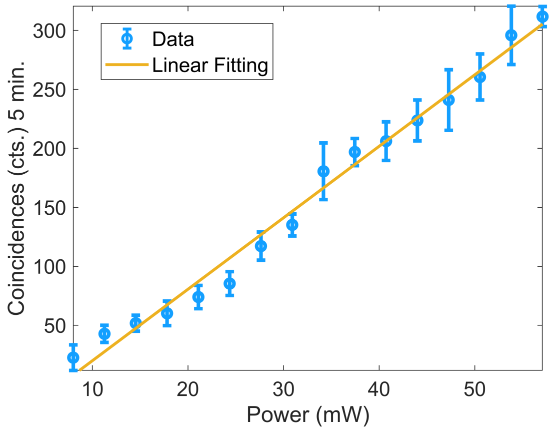

Fig. S6: Power dependence of the SPDC rate measured in forward direction.

S6 Photon-pair spectrum

We characterize the spectrum of the photon-pairs using a correlation-based spectroscopic measurement method [7], [8]. Here, the photon pairs are sent through a long, dispersive medium before coincidence detection. The different group velocities for photons with different frequencies effectively lead to different arrival times at the detectors, which can be tracked by means of the coincidence histogram. Provided the dispersion of the propagating medium is known, this varying time delay can be mapped to a wavelength. To perform this measurement, we replace the multimode fiber by 2 km of SMF28 single mode fiber, and use single-mode fiber coupled single-mode fiber coupled super-conducting nanowire single photon detectors (SNSPD). Fig. S7 shows a representative spectrum for excitation of the metasurface with the linear transmission spectrum shown in Fig. S7 for excitation at $\lambda_p = 786.5\,\text{nm}$. The resulting SPDC spectrum shows a peak at the degenerated SPDC wavelength with a narrow linewidth, as defined by the narrow-band resonances of the metasurface.

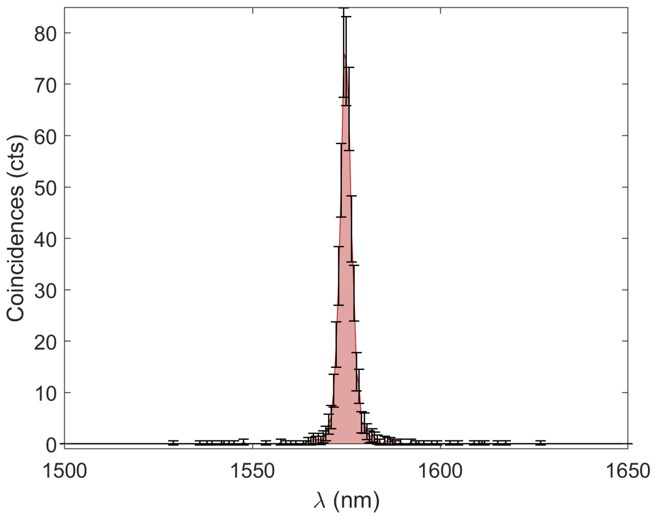

Fig. S7: Representative SPDC spectrum for excitation at 786.5 nm. The SPDC peak appears at the degenerate SPDC wavelength and has a narrow bandwidth, defined by the resonance of the metasurface.

S7 Raw data angularly tunable photon-pair generation

In this section we show the raw data for the angularly tunable photon-pair generation experiments reported in Fig. 5 of the main text. In these measurements, the movable beam-block placed in the forward arm (see Fig. S1 and Fig. 4(a) of the main text) is moved in steps of 25 μm in the back-focal plane of the collection lens. For each beam-block displacement d, a coincidence histogram was recorded. In Fig. S8 an exemplary plot of the coincidence histogram depending on the beamblock displacement is shown. It is clearly visible that around the positions of ± 1 mm the number of coincidences drops, as the beamblock crosses the angular emission direction of one of the two photons of the pair. Considering that the beamblock is placed in the back-focal plane, i.e. at focal distance f behind the collection lens, the emission angle ϑ associated to a certain transverse position of the beamblock is directly obtained from $\vartheta = \text{atan}(d/f)$. For a plane-wave with momentum $k_0 = 2\pi/\lambda$ this corresponds to a transverse momentum of $k_y = \sin(\vartheta)k_0$.

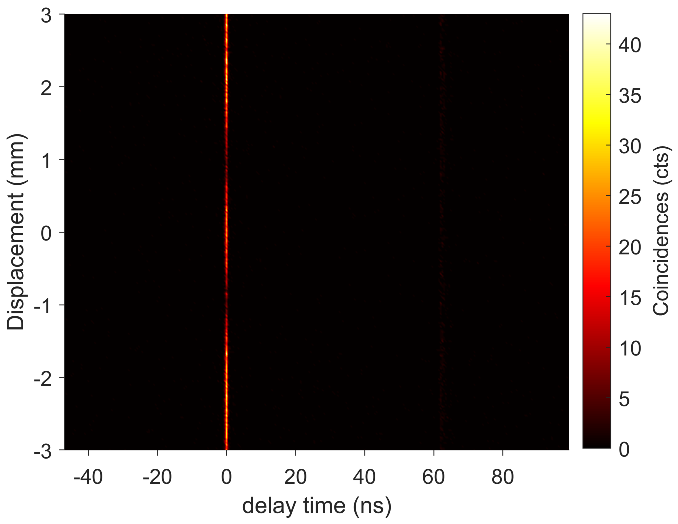

Fig. S8: Evolution of coincidence histograms for excitation with $\lambda_p = 782.7$ nm, power $P = 92$ mW for substrate side excitation and copropagating (forward) collection geometry. The exposure time for each histogram is $t = 60$ s.

From this series of histograms, the coincidence rate is extracted by integrating the time bins around the zero time-delay position. This procedure is carried out for various excitation wavelengths and for copropagating (forward) and counterpropagating detection. In Fig. S9, all coincidence rates measured for copropagating detection and different excitation wavelengths are summarized. The measured data points are marked by orange circles. Since these coincidences are subject to statistical noise, we use a moving average filter with window size ± 2 data points when plotting the graph connecting all data points (blue lines). These curves are shown as surface plot in Fig. 5(b,c) of the main text. The raw data for the counterpropagating measurements is shown in Fig. S10 and processed analogously.

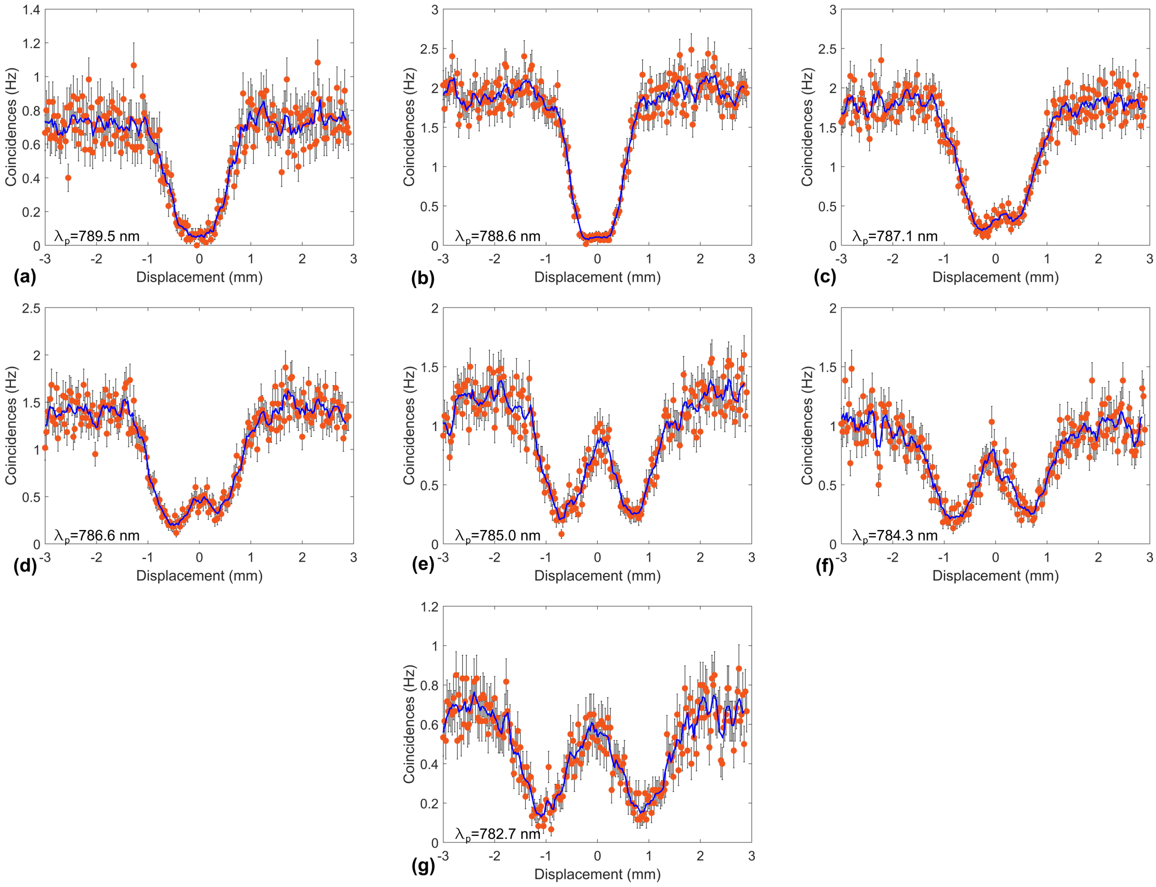

Fig. S9: Coincidence rate depending on beamblock position for copropagating (forward) collection geometry and varying excitation wavelengths: (a) $\lambda_p = 789.5\,\mathrm{nm}$, (b) $\lambda_p = 788.6\,\mathrm{nm}$, (c) $\lambda_p = 787.1\,\mathrm{nm}$, (d) $\lambda_p = 786.6\,\mathrm{nm}$, (e) $\lambda_p = 785.0\,\mathrm{nm}$, (f) $\lambda_p = 784.3\,\mathrm{nm}$ and (g) $\lambda_p = 782.7\,\mathrm{nm}$. Orange dots mark the measured coincidence rate, blue solid lines are moving average fits and error bars due to statistical uncertainty are shown in black.

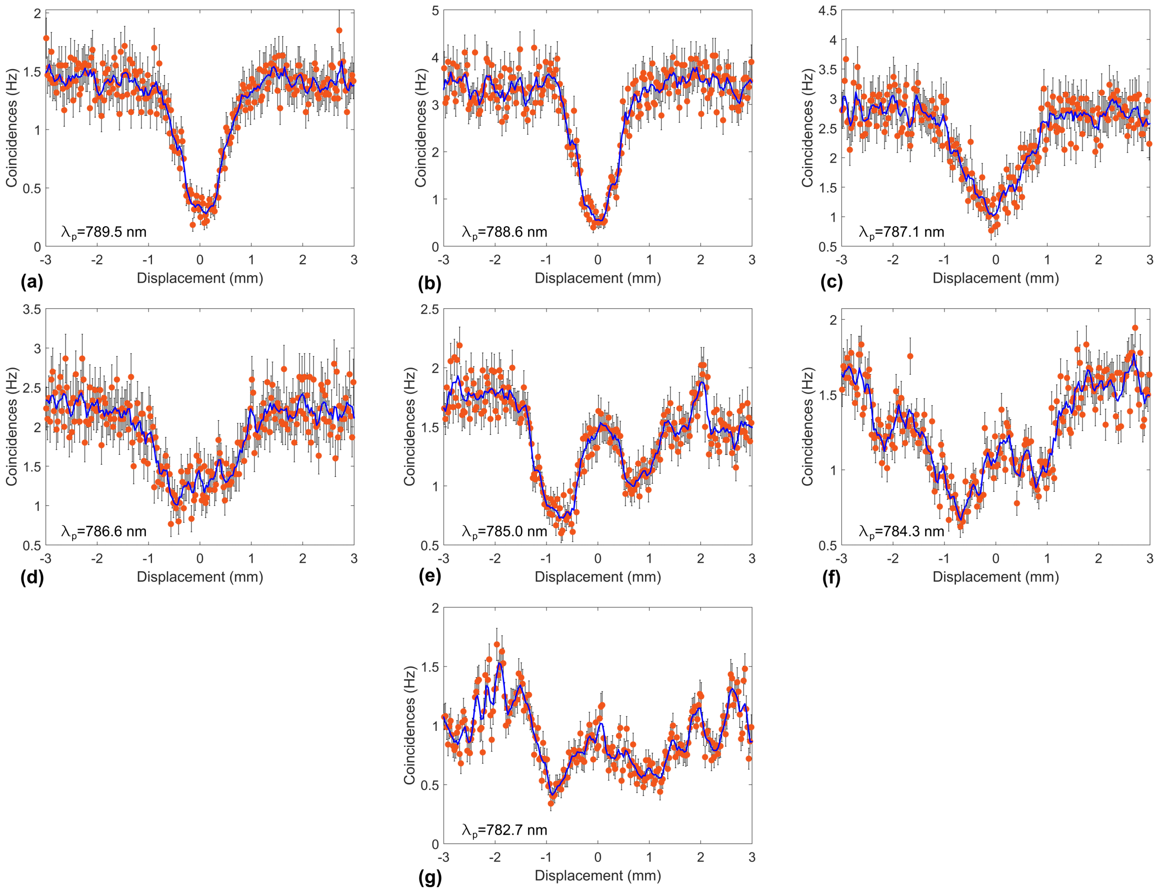

Fig. S10: Coincidence rate depending on beamblock position for counterpropagating collection geometry and varying excitation wavelengths: (a) $\lambda_p = 789.5\,\text{nm}$, (b) $\lambda_p = 788.6\,\text{nm}$, (c) $\lambda_p = 787.1\,\text{nm}$, (d) $\lambda_p = 786.6\,\text{nm}$, (e) $\lambda_p = 785.0\,\text{nm}$, (f) $\lambda_p = 784.3\,\text{nm}$ and (g) $\lambda_p = 782.7\,\text{nm}$. Orange dots mark the measured coincidence rate, blue solid lines are moving average fits and error bars due to statistical uncertainty are shown in black.

References

[1] S. S. Wang and R. Magnusson, "Theory and applications of guided-mode resonance filters," *Applied Optics*, vol. 32, no. 14, p. 2606, 1993. DOI: 10.1364/AO.32.002606.

[2] S. S. Wang, R. Magnusson, J. S. Bagby, and M. G. Moharam, "Guided-mode resonances in planar dielectric-layer diffraction gratings," *JOSA A*, vol. 7, no. 8, pp. 1470–1474, Aug. 1, 1990. DOI: 10.1364/JOSAA.7.001470.

[3] J. P. Hugonin and P. Lalanne, "RETICOLO software for grating analysis," *arxiv preprint arxiv:2101.00901*, 2023. DOI: 10.48550/arXiv.2101.00901.

[4] S. Lee and K. H. Kim, "Analytical Determination of Multilayer Waveguide Modes," *Journal of the Korean Physical Society*, vol. 51, no. 1, pp. 104–106, 2007. DOI: 10.3938/jkps.51.104.

[5] M. F. Limonov, M. V. Rybin, A. N. Poddubny, and Y. S. Kivshar, "Fano resonances in photonics," *Nature Photonics*, vol. 11, no. 9, pp. 543–554, 9 2017. DOI: 10.1038/nphoton.2017.142.

[6] T. T. Hoang, Q. M. Ngo, D. L. Vu, and H. P. T. Nguyen, "Controlling Fano resonances in multilayer dielectric gratings towards optical bistable devices," *Scientific Reports*, vol. 8, no. 1, p. 16 404, 1 2018. DOI: 10.1038/s41598-018-34787-9.

[7] A. Valencia, M. V. Chekhova, A. Trifonov, and Y. Shih, "Entangled Two-Photon Wave Packet in a Dispersive Medium," *Physical Review Letters*, vol. 88, no. 18, p. 183 601, 2002-04-17. DOI: 10.1103/PhysRevLett.88.183601.

[8] C. Okoth, A. Cavanna, T. Santiago-Cruz, and M. V. Chekhova, "Microscale Generation of Entangled Photons without Momentum Conservation," *Physical Review Letters*, vol. 123, no. 26, p. 263 602, 2019. DOI: 10.1103/PhysRevLett.123.263602.
